# Stability of Zika Virus Antibodies in Specimens from a Retrospective Serological Study

**DOI:** 10.4269/ajtmh.21-0564b

**Published:** 2021-07-26

**Authors:** R. Tedjo Sasmono, Edison Johar, Benediktus Yohan, Chairin Nisa Ma’roef, Amin Soebandrio, Khin SA Myint, Paul Pronyk, Sri Rezeki Hadinegoro, Elizabeth Jane Soepardi, Alain Bouckenooghe, William Hawley, Ronald Rosenberg, Ann M. Powers

**Affiliations:** Eijkman Institute for Molecular Biology Jakarta, Indonesia E-mails: sasmono@eijkman.go.id, edisonjohar@eijkman.go.id, yohan@eijkman.go.id, nami@eijkman.go.id, aminsoebandrio@eijkman.go.id, and myint@eijkman.go.id; UNICEF Indonesia Jakarta, Indonesia E-mail: ppronyk@unicef.org; Cipto Mangunkusumo Hospital Universitas Indonesia Jakarta, Indonesia E-mail: shadinegoro46@gmail.com; Ministry of Health of the Republic of Indonesia Jakarta, Indonesia E-mail: ejanesoepardi@gmail.com; Sanofi Pasteur Lyon, Rhone-Alpes, France E-mail: alainbouck@gmail.com; Centers for Disease Control and Prevention Atlanta, Georgia E-mail: byh0@cdc.gov; Centers for Disease Control and Prevention Fort Collins, Colorado E-mails: dcx7@cdc.gov and ann.powers@cdc.hhs.gov Published online July 26, 2021.

Dear Sir,

We would like to respond to the letter by Zhang and others regarding our study published in the *American Journal of Tropical Medicine and Hygiene*.^1^ Our serum samples were all stored in annually calibrated –80°C freezers. The samples were only thawed once. In general, antibodies are known to remain stable in frozen storage over lengthy periods. There are numerous publications regarding antibody stability during storage,^2–6^ and we believe that all antibodies, including antibodies against Zika virus, will remain stable during storage.

Our study also re-assayed the presence of anti-dengue IgG antibodies in the samples. In our previous study,^7^ we measured dengue IgG antibody using indirect ELISA, and in the current study^1^ we tested for antibodies in the same samples using a plaque reduction neutralization test. Overall good concordance of results between the two methods was observed (not shown), supporting the stability of antibodies in our samples.

The characterization of anti-Zika virus antibody stability during storage may be useful, but this was not the focus of our study.
